# Endothelial Nitric Oxide Synthase Reduces Crescentic and Necrotic Glomerular Lesions, Reactive Oxygen Production, and MCP1 Production in Murine Lupus Nephritis

**DOI:** 10.1371/journal.pone.0064650

**Published:** 2013-05-31

**Authors:** Gary S. Gilkeson, Ahmad K. Mashmoushi, Phillip Ruiz, Tiffany N. Caza, Andras Perl, Jim C. Oates

**Affiliations:** 1 Division of Rheumatology and Immunology, Department of Medicine, Medical University of South Carolina, Charleston, South Carolina, United States of America; 2 Medical Service, Ralph H. Johnson VA Medical Center, Charleston, South Carolina, United States of America; 3 Transplant Laboratories and Immunopathology, Department of Surgery and Pathology, University of Miami Miller School of Medicine, Miami, Florida, United States of America; 4 Division of Rheumatology, Department of Medicine, Upstate Medical University, State University of New York, Syracuse, New York, United States of America; University of Texas Medical Branch, United States of America

## Abstract

Systemic lupus erythematosus, in both animal models and in humans, is characterized by autoantibody production followed by immune complex deposition in target tissues. Ensuing target organ damage is modulated by reactive intermediates, including reactive nitrogen and oxygen species, through as of now incompletely understood mechanisms. Endothelial nitric oxide synthase is known to impact vascular reactivity; however its impact on reactive intermediate production and inflammatory renal disease is less well defined. In this study, we assessed the impact of endothelial nitric oxide synthase (eNOS) on disease in lupus prone MRL/lpr mice. Mice lacking eNOS developed earlier more severe disease with decreased survival. eNOS deficient mice died sooner and developed significantly more glomerular crescents, necrosis, inflammatory infiltrates and vasculitis, indicating a role for eNOS in modulating these renal lesions. Immune complex deposition was similar between groups, indicating the impact of eNOS is distal to antibody/complement glomerular deposition. Urinary nitric oxide production was decreased in the eNOS deficient mice, while proteinuria was increased. Urinary monocyte chemotactic protein-1 was also increased in the knockout mice. CD4+ T cells from MRL/lpr mice demonstrated mitochondrial hyperpolarization, increased nitric oxide and superoxide production and increased calcium flux compared to B6 control mice. Deficiency of eNOS resulted in decreased nitric oxide and mitochondrial calcium levels but had no effect on mitochondrial hyperpolarization. Renal cortices from MRL/lpr mice that are eNOS deficient demonstrated increased superoxide production, which was blocked by both nitric oxide synthase and NADPH oxidase inhibitors. These studies thus demonstrate a key role for eNOS in modulating renal disease in lupus prone MRL/lpr mice. The impact appears to be mediated by effects on superoxide production in the kidney, impacting downstream mediators such as monocyte chemotactic protein-1. These results suggest that modulation of eNOS may be a novel therapeutic approach to treating lupus nephritis.

## Introduction

Systemic lupus erythematosus (SLE) is an autoimmune disease characterized by autoantibody production that leads to enhanced innate immune responses in affected tissues [1]. Reactive oxygen species such as superoxide (SO) and hydrogen peroxide can modify enzymes altering their function in a process known as reduction-oxidation (redox) signaling. This process is critical in many innate immune responses [2]. Nitric oxide (NO) is a membrane-permeable free radical that is formed by any of the three isoforms of nitric oxide synthase (NOS), using arginine and oxygen as substrates [3]. NO is implicated in many physiological as well as pathological processes; this dual effect of NO reflects differences in local levels of NO production in addition to the presence of other reactive intermediates [4].

Lupus patients often display a phenotype of eNOS dysfunction with reduced endothelium-dependent vasodilation [5]. The mechanism behind this finding is unclear but is consistent with reports of low levels of eNOS expression observed in kidney biopsy specimens from patients with lupus nephritis [6], [7]. eNOS plays a vital role in endothelial cell physiology. Several studies employing eNOS knockout murine models demonstrated the role of eNOS-NO in the maintenance of normal blood pressure, coagulation, and leukocyte adhesion [8]. On the other hand, eNOS-NO may play a role in T cell activation and clearance. Several studies showed that NO induces mitochondrial membrane hyperpolarization (MHP) and biogenesis, increases [Ca^2+^ ] in the cytosol and mitochondria of normal T cells, and recapitulates the enhanced CD3/CD28-induced Ca^2+^ fluxing of lupus T cells [9].

Both iNOS and eNOS are widely expressed in the kidneys; nNOS expression is limited to the macula densa region. Previous murine studies in our laboratory suggest that iNOS may contribute to glomerular pathology in lupus nephritis due to its ability to produce reactive oxygen species when uncoupled [10]. On the other hand, the functional role of eNOS in lupus is still unknown. The decline of eNOS expression in lupus may indicate a protective role of this enzyme. To investigate the role of eNOS in lupus, MRL/MpJ-*FAS^lpr^* (MRL*/lpr*) NOS3 knockout mice (NOS3−/−) were derived to determine the effect on the lupus disease, reactive oxygen and nitrogen production, chemokine production, and T cell biology.

## Materials and Methods

### Ethics Statement

This study was carried out in strict accordance with the recommendations in the Guide for the Care and Use of Laboratory Animals of the National Institutes of Health. The Ralph H. Johnson VA Institutional Animal Care and Use Committee approved all procedures.

### General Protocol

MRL/lpr mice lacking NOS3 (NOS3−/−) were generated as described below. Before sacrifice, individual mice were placed on a 24-hour low nitrate+nitrite (NO_X_) diet (Zeigler Brothers, Gardners PA) with distilled water and placed in metabolic cages for an additional 24 hours on the same diet/water for urine collection. At 16–20 weeks of age (after the onset of proteinuria ≥100 mg/dl by dipstick, but before premature death from renal disease), mice were sacrificed with age-matched littermate pairs (NOS3+/+) for harvest of renal and spleen tissue and blood. Renal cortical tissue from one kidney was removed, snap frozen in liquid nitrogen and stored at −80°C for analysis. The contralateral kidney was immediately fixed in paraformaldehyde and stained with hematoxylin and eosin before examination by a pathologist (PR) who was masked to the treatment group. Blood was centrifuged after clotting, and serum was stored in aliquots at −80°C for analysis. Spleens were suspended in Dulbecco’s modified Eagle medium with 20% fetal bovine serum and shipped on ice overnight for flow cytometry analysis. NOS3−/− mice died much earlier than NOS3+/+ mice, necessitating earlier sacrifice for study of tissue (14 weeks) than is our general practice with MRL/lpr mice (20–22 weeks).

### Generation of the NOS3−/− and NOS2−/− MRL/lpr Mice

MRL/MpJ-*FAS^lpr^* (MRL*/lpr*) mice were purchased from Jackson Laboratory (Bar Harbor, ME) and housed under specific pathogen–free conditions in the animal research facility at the Ralph H. Johnson Veterans Affairs Medical Center in Charleston, SC. B6.129P2-Nos3<tm1Unc>/J mice purchased from Jackson Laboratories were bred onto the MRL/MpJFas/lpr background. These NOS3−/− mice were backcrossed nine times to MRL/lpr mice. Speed congenics techniques were used as previously described to ensure backcross of MRL/lpr susceptibility loci to the NOS3−/− mice [11]. Fifteen genetic susceptibility loci and the NOS3−/− genotype were confirmed by polymerase chain reaction [12], [13]. MRL/lpr NOS3+/− mice were bred to generate wild type MRL/lpr (NOS3+/+) and MRL/lpr NOS3−/− (NOS3−/−) littermates for the subsequent studies. To prevent the influence of genetic drift on the glomerulonephritis phenotype, NOS3−/− male mice were periodically backcrossed with the MRL/lpr female mice from Jackson Laboratories. NOS2−/− mice were generated as previously described [14].

### Urine Nitrate+Nitrite (NO2/NO3) Albumin and Anti-double Stranded DNA Antibody (dsDNA) Assays

After 24 hour urine samples were collected in an antibiotic cocktail, urine NO_2_/NO_3_ was measured using a chemiluminescence detector as described [10], [14]. Standard enzyme-linked immunosorbent assay (ELISA) techniques were used to detect urine albumin using the published methods of Sekine et al. and reported as mg of urine albumin/mouse/day (8). Serum dsDNA antibody levels were measured as described [15].

### Qualitative Glomerular Scores

At the end of treatment, mice were sacrificed for harvest of tissue. One kidney was removed from each mouse and immediately fixed in paraformaldehyde and stained with hematoxylin and eosin before examination by a pathologist (PR) who was masked to the treatment group. Pathologic changes of renal disease activity were graded qualitatively as previously published (8). Glomeruli were graded for hypercellularity (0–4), mesangial expansion (0–4), hyperlobulation (0–4), chronic inflammation (0–4), dilatation (0–4), necrosis (0–4), hyperlobularity (0–4), crescent formation (0–4), epithelial cell reactivity (0–4), thrombi in loops (0–4), vasculitis (0–4), thickened membranes (0–4), and neutrophil debris (0–4). Scores for crescent formation and necrosis are doubled, consistent with the severity of these lesions. Scores were additive, with the most common elements seen in this model being hypercellularity, crescent formation, and necrosis.

### Immunofluorescence Detection of IgG and C3 in Glomerular Tissue

Frozen kidney tissue was fixed in acetone and stained FITC-conjugated monoclonal IgG antibody to C3 or IgG (Cappel, MP Biomedicals, Solon, OH) as described [14]. A Nikon Eclipse 80i microscope and camera (DS-Qi1Mc) and Nikon NIS-Element Basic Research software (version 3.07) were used to acquire and analyze pixel intensity and area of fluorescent stain within glomeruli. Pixel density from ten glomeruli per specimen was analyzed, and the average intensity of the readings for each glomerulus was reported.

### Urine MCP-1

Urine collected as above was analyzed for MCP-1 using ELISA kits (eBioscience, San Diego, CA), following the manufacturer’s instructions. Urine was diluted 1∶10 to reduce interference from the urine matrix [16]. Measurements were performed in triplicate.

### Isolation of Mouse Thymocytes and Spleen Cells

Single-cell suspensions of spleen cells were prepared by disrupting spleen onto 70 µm filters on top of 50 mL conical tubes and rinsing cells through by addition of supplemented DMEM medium (DMEM base, 10% fetal bovine serum, 2 mM L-glutamine, 100 U/mL penicillin, 100 µg/mL streptomycin, 0.25 µg/mL amphotericin B, 1 mM sodium pyruvate, 1% MEM non-essential amino acid solution). Cells were centrifuged at 1200×g for 10 minutes, and pellets were resuspended in ACK lysis buffer (150 mM NH4Cl, 1 mM KHCO3, and 1 µM EDTA) for 5 minutes to remove red blood cells. Medium was added to stop the lysis reaction and cells were centrifuged, counted, and resuspended at 2×10^6^ cells/mL for analysis.

### Flow Cytometric Analysis of Metabolic Indicators

Methods for analysis of metabolic markers in CD4+ spleen cells were derived from previously published assays and adapted for murine cells [17]. For metabolic labeling, cells were stained in DMEM medium at a density of 1×10^6^ cells/ml with cell-permeable metabolic dyes at 37°C for 30–120 minutes, followed by surface staining with PE Cy7 conjugated CD3 (17A2), PerCP conjugated CD4 (GK1.5), APC Cy7 conjugated CD8a (53–6.7), PE conjugated CD11b (M1/70), APC conjugated CD11c (N418), and Alexa Fluor 700 conjugated CD19 (6D5) antibodies for 30 minutes at 4°C. All antibodies were obtained from Biolegend (San Diego, CA). After staining with antibodies, cells were washed twice in cold medium, and maintained on ice until flow cytometry measurement. Samples were analyzed on a Becton-Dickinson LSR-II flow cytometer, equipped with 20 mW solid-state Ng-YAG, 20 mW argon, 10 mW diode pumped solid state yellow-green, and 16 mW helium-neon lasers (BD, Franklin Lakes, NJ), with 50,000 events per sample collected. Data was evaluated using Flow Jo cytometry analysis software (TreeStar corporation, Ashland, OR).

Cell-permeable metabolic indicators were used for measurement of NO, peroxynitrite, mitochondrial mass, mitochondrial Ca2+, cytosolic Ca2+, hydrogen peroxide, and superoxide production. DAF-FM (1 µM) was used to measure NO production. Diaminorhodamine-4M (DAR-4M, 1 µM) was used to evaluate peroxynitrite production, a byproduct of NO. DiOC6 (4 nM) was utilized as mitochondrial mass indicators. Calcium stores were measured by fluorescence of Rhod-2 AM (1 µM) and Fluo-3 AM (1 µM). Hydroethidine was used as an indicator for superoxide production (HE, 1 µM) and DCF-DA (1 µM) for superoxide production. Dead cells were excluded from analysis by Annexin V (10 µg/mL) and PI staining (50 µg/mL). DAF-FM, DiOC6, HE, DCF-DA, Rhod-2, Fluo-3, and PI were obtained from Invitrogen-Molecular Probes (Carlsbad, CA). Annexin V-FITC was obtained from BD Transduction Laboratories (Franklin Lakes, NJ). DAR-4M was from Calbiochem (San Diego, CA).

### Lucigenin Assay to Determine Renal Cortical SO Production

Mouse renal cortices (at least three from each genotype) were homogenized in a detergent-free lysis buffer (pH 7.4) containing 150 mM NaCl, 50 mM Tris, 25 mM ethyleneglycoltetraacetic acid (EGTA), 25 mM ethylenediaminetetraacetic acid (EDTA), with a protease inhibitor and phosphatase inhibitor cocktail. Freshly prepared homogenates were then used to determine spontaneous SO production using lucigenin-enhanced chemiluminescence as described [18], [19]. A total volume of 100 µl of homogenate was incubated for 30 minutes at 37°C with the following: no treatment, DPI (50 µM), allopurinol (50 µM), L-NMMA (50 µM), rotenone (20 µM), mefenamic acid (20 µM), 2-thenoyltrifluoroacetone (10 µM), antimycin A (10 µM), and o-napthoflavone (1 µM). Incubated samples (50 µl) were placed in a 24-well reading plate containing 25 µM lucigenin (Sigma) for the detection of SO in a final volume of 500 µl of Krebs solution buffered with 10 mM HEPES-NaOH (pH 7.4). Readings for each sample were performed using Luminoskan luminometer at 37°C in triplicates and normalized to protein level measured by protein assay (Bio-Rad) after background subtraction.

### Statistics

Raw values for lucigenin fluorescence were normalized in each experiment to control values from MRL/lpr mice with no inhibitors present. At least three experiments were performed, with three replicates each. Normalized values were compared between genotypes and inhibitor conditions. Comparisons between groups were made using Student’s t-test if data were normally distributed or Wilcoxon rank sum if they were not normally distributed. Correlations were performed using Spearman or Pearson correlation depending on the distribution of the data. Data were reported as the median (interquartile range (IQR)).

## Results

### Mice

22 NOS3−/− and 24 NOS3+/+ MRL/lpr mice were available for study. The original protocol called for sacrifice at close to 20 weeks of age. It became evident quite early in the study that NOS3−/− mice died as early as six weeks of age in comparison to an historical average of 20–22 weeks in wild type MRL/lpr mice in our laboratory. While the protocol was not designed as a formal survival study, the number of mice who either died in their cages or were moribund (and thus euthanized humanely) before being euthanized for pathology studies was much higher in the NOS3−/− mice versus the NOS3+/+ mice (20/104 (19%) vs. 4/84 (5%)). During the urine collection just before death, NOS3−/− mice had greater proteinuria than the NOS+/+ mice (7±9 vs. 0.6±0.8 mg/mouse/day). Due to these premature deaths, urine and serum were collected on more mice than were available for pathologic study. To increase the number of mice for pathologic study, the protocol was modified so that mice with proteinuria ≥100 on dipstick were sacrificed. Using this new protocol, ten NOS3−/− and 11 NOS3+/+ mice were available for pathology studies. This number was within the initial sample size calculation to show significant differences between groups with a power of 0.8 and an α of 0.05.

### Urine NO_X_ Levels were Lower, and Albuminuria was Greater in NOS3−/− Mice

**Figure 1:**
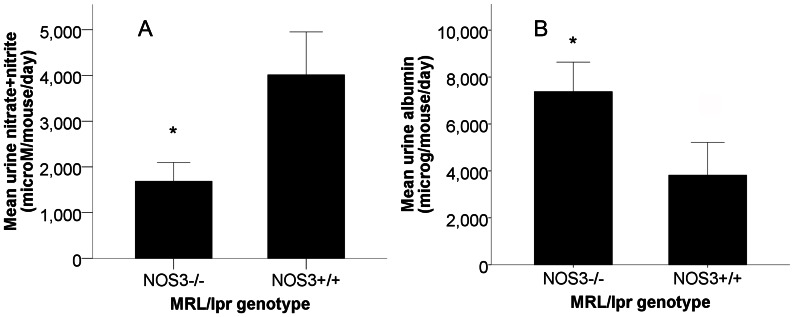
Urine albumin is greater, while systemic nitric oxide production is reduced in NOS3−/− mice. Urine from MRL/lpr NOS3−/− (n = 22) and MRL/lpr NOS3+/+ (n = 24) mice was analyzed during active disease for albumin (mg/mouse/day) and nitrate+nitrite (µmoles/mouse/day). Urine nitrate+ nitrite (A) was significantly lower in NOS3−/− mice, while urine albumin (B) was greater in NOS3−/− mice. * p<0.02 each.

To determine the effect of the NOS3−/− genotype on systemic NO production, urine was collected for analysis every two weeks. The urine collected just prior to sacrifice or early death was analyzed for total nitrate and nitrite content in a 24-hour collection. Urine NOX was significantly lower in NOS3−/− mice (Figure 1A). This finding suggests that eNOS-derived NO is a significant contributor to systemic NO production in early active disease. To determine the effect of lack of functional eNOS on renal disease, proteinuria as a marker of glomerulopathy was determined. During active disease, urine albumin levels were significantly higher among the NOS3−/− compared to the wild type NOS3+/+ mice (Figure 1B). This difference suggests a glomerulo-protective role of eNOS in this model.

### Glomerular Pathology Indicates Higher Hypercellularity, Necrosis, Crescent Formation, Tubular Inflammation, and Vasculitis in Mice Lacking NOS3

**Figure 2:**
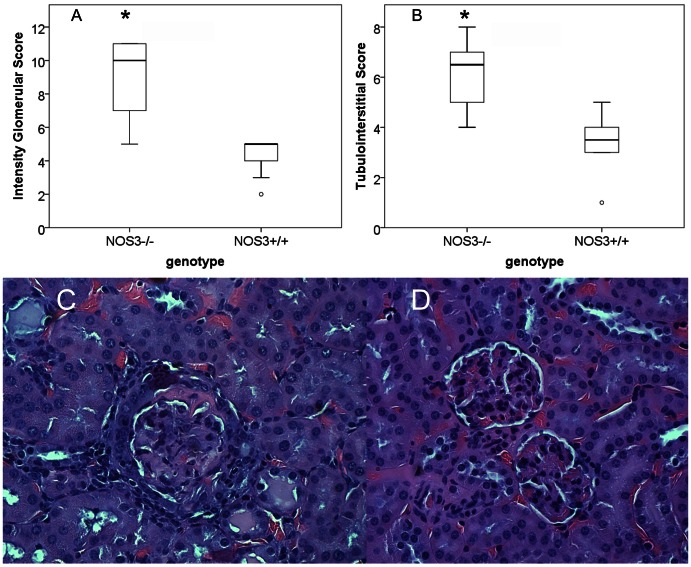
Glomerular and tubulointerstitial pathology is greater in NOSe−/− mice. Kidney cortical tissue from MRL/lpr NOS3−/− and MRL/lpr NOS3+/+ mice (n = 10) was fixed and stained and analyzed for glomerular and tubulointerstitial scores. Both glomerular (A) and tubulointerstitial (B) total scores were greater in NOS3−/− mice. Representative images of glomeruli from 13 week old NOS3−/− (C) and NOS3+/+ (D) mice demonstrate the differences in crescentic and necrotic lesions between the two groups. * p<0.01.

To determine the effect of the NOS3−/− genotype on glomerular pathology, kidneys were harvested at the time of sacrifice (in NOS3−/− and WT littermate pairs), sectioned, stained with H&E, and scored for pathologic changes. Total glomerular and tubulointerstitial scores were significantly greater in NOS3−/− mice (Figure 2). Significant differences in individual elements of the glomerular scores were observed. For instance, focal hypercellularity, necrosis, crescents, chronic inflammation, tubular casts, and vasculitis scores were significantly greater in NOS3−/− compared to NOS3+/+ controls (Figure 2C and 2D and Table 1). These results suggest that NOS3 is important in three potentially distinct processes in the setting of proliferative lupus nephritis. First, eNOS appears to protect from vascular injury leading to glomerular necrosis and crescent formation. Second, eNOS appears to protect from cellular migration and proliferation in the kidney. Third, NOS3 appears to protect from medium vessel vasculitis. Whether this protection is related to changes in humoral autoimmunity or innate and cellular immune activation distal to immune complex deposition in the kidney is not clear from pathology alone.

**Table 1:** Median scores for individual pathologic findings with p<0.05 in NOS3−/− versus NOS3+/+ mice.

Pathologic finding	NOS3 genotype	median (IQR)	p value
Focal hypercellularity	−/−	3 (3–3)	<0.001
	+/+	2 (2–2)	
Necrosis	−/−	4 (0–4)	0.001
	+/+	0 (0–0)	
Crescents	−/−	4 (0–4)	0.001
	+/+	0 (0–0)	
Tubular casts	−/−	1 (0–2)	0.01
	+/+	0 (0–0)	
Chronic inflammation	−/−	2 (1–3)	0.001
	+/+	1 (1–1)	
Vasculitis	+/+	1 (1–1)	<0.001
	+/+	0 (0–0)	

IQR = interquartile range.

−/− = MRL/lpr NOS3−/−.

+/+ = MRL/lpr NOS3+/+.

### Differences in Glomerular Pathology were not Reflected in Differences in either Glomerular IgG and C3 Immunofluorescence or Serum dsDNA Levels

To determine if the differences in histopathology observed above were in part due to the effect of eNOS on T cell function and resulting pathogenic humoral autoimmunity, the extent of IgG and C3 deposition in the glomeruli of mice from both groups was determined. Kidneys were graded for glomerular IgG and C3. There were no significant differences in either measure between groups (Figure S1). Similarly, serum anti-dsDNA antibody levels were no different between groups (Figure S2). These results suggest that differences in pathology observed between the two groups are due to differences in innate and cellular immunity distal to immune complex deposition.

### T cell NO Production and Mitochondrial Calcium are Reduced in NOS3−/− Mice

**Figure 3:**
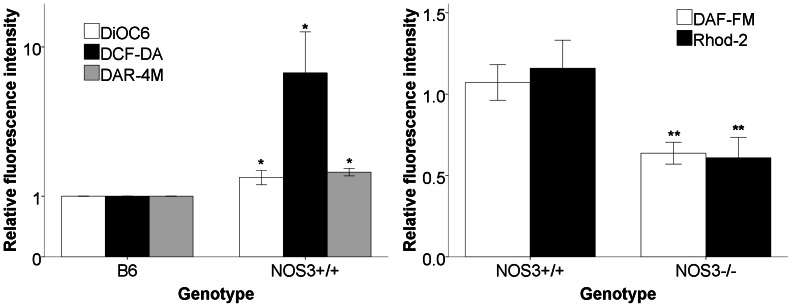
CD4+ spleen cells from MRL/lpr mice have greater mitochondrial transmembrane potential, reactive oxygen production, and reactive nitrogen production, while NOS3−/− mice have reduced nitric oxide and mitochondrial calcium production. Spleen cells were isolated from B6 (n = 8) and NOS3+/+ (n = 7) mice (A) and NOS3−/− (n = 7) and NOS3+/+ (n = 7) mice (B) and analyzed for markers of mitochondrial mass, mitochondrial transmembrane potential, calcium flux, nitric oxide, peroxynitrite, reactive oxygen species, and glutathione by flow cytometry and reported relative to controls. MRL/lpr CD4+ cells had greater markers of transmembrane potential (DiOC6), reactive oxygen production (DCF-DA), and peroxynitrite production (DAR-4M) compared to B6 controls (A). NOS3−/− CD4+ T cells exhibited lower levels of NO (DAF-FM) and mitochondrial calcium production (Rhod-2) (p<0.05) but no differences in markers of mitochondrial mass and transmembrane potential (not shown). All data were reported as fluorescence intensity relative to values for B6 mice analyzed on the same day. * p<0.05 for NOS3+/+ vs. B6, ** p<0.05 for NOS3−/− vs. NOS3+/+.

To determine the effect of deficient NOS3 on T cell biology, spleen cells from B6, MRL/lpr NOS3+/+, MRL/lpr NOS3−/− and MRL/lpr NOS2−/− mice were removed at sacrifice and stained for markers of cell type, intracellular glutathione, mitochondrial potential, mitochondrial mass, oxidation, nitric oxide, calcium, and cell death. Consistent with observations in humans with lupus [20], CD4+ T cells from MRL/lpr mice had increased measures of mitochondrial potential (DiOC6), intracellular reactive oxygen (DCF-DA), and nitric oxide and peroxynitrite (DAR-4M) (Figure 3A). When the MRL/lpr NOS3−/− and MRL/lpr NOS3+/+ CD4+ T cells were compared, markers of NO production were reduced (Figure 3B) in a fashion not seen in MRL/lpr NOS2−/− mice (data not shown), suggesting that increased CD4+ T cell NO production in these mice is at least partially eNOS-dependent and is not iNOS-dependent. Mitochondrial calcium is reduced in the NOS3−/− CD4+ T cells, suggesting a potential effect on calcium-mediated signaling in mitochondria. However, the increase seen in mitochondrial membrane potential in MRL/lpr NOS3+/+ mice was not reduced significantly in the MRL/lpr NOS3−/− mice, suggesting alternate signaling pathways for this phenomenon. This, combined with the lack of significant changes in anti-dsDNA antibody production in the NOS3−/− mice and lack of significant differences in glomerular IgG and C3 deposition, suggests that the pathologic effects of eNOS dysfunction in this model is distal to autoantibody production and immune complex deposition and may be important in tissue innate immune responses to immune complexes.

### Superoxide Production in Renal Cortical Tissue was Modulated by eNOS and Catalyzed by NOS and Nicotinamide Adenine Dinucleotide Phosphate (NADPH) Oxidase (NOX)

**Figure 4:**
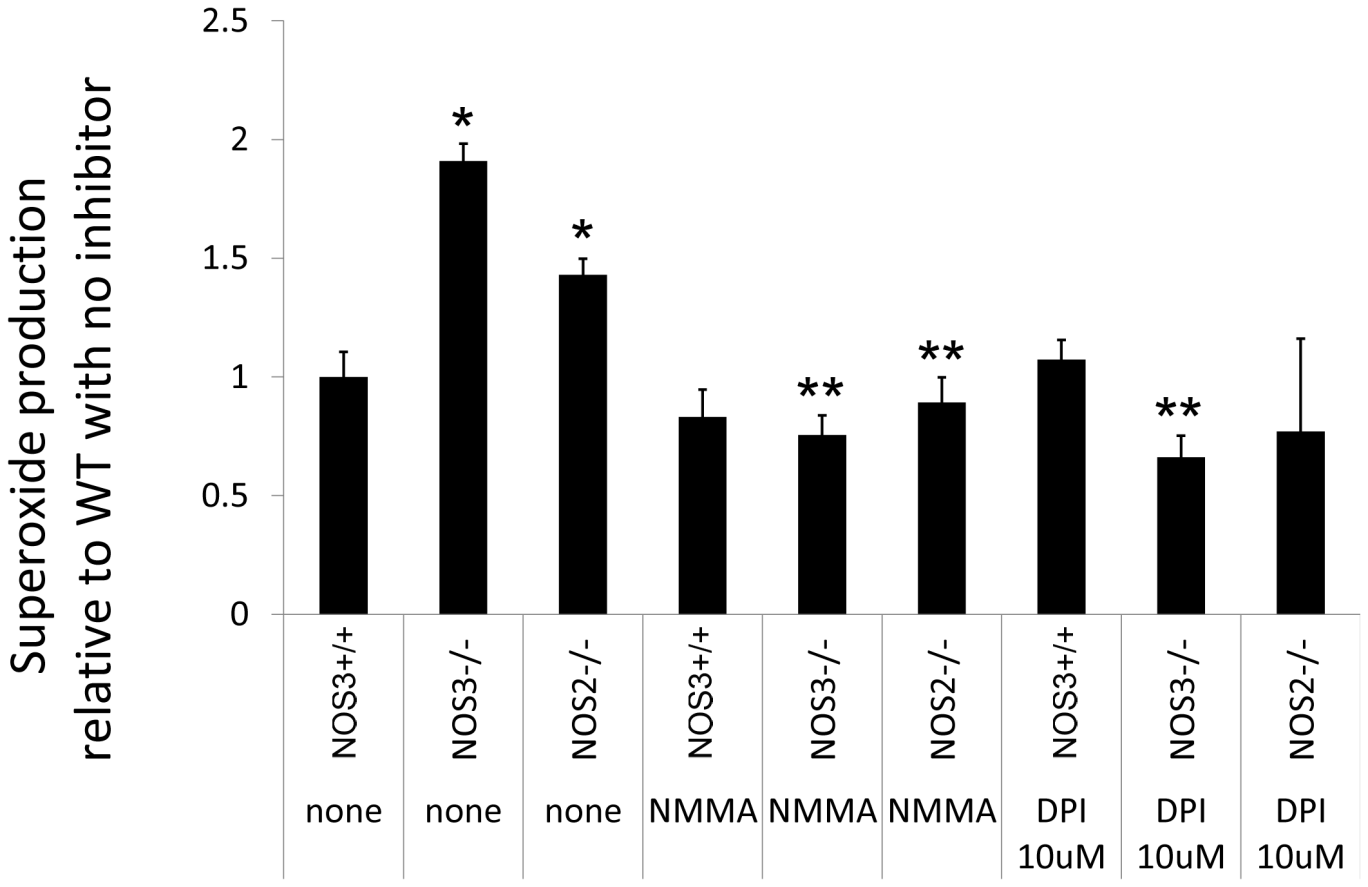
Renal cortex from MRL/lpr mice produces more superoxide (SO) in the absence of NOS3 and NOS2 and less superoxide in the presence of NOS and NOX inhibitors. Renal cortical tissue from MRL/lpr NOS3−/− (NOS3−/−), MRL/lpr NOS2−/− (NOS2−/−) and MRL/lpr NOS3+/+ littermates was examined for SO production in the presence of inhibitors of known enzyme sources of SO production. Only the significant results (p<0.05) were reported. Renal cortex from mice lacking NOS3 or NOS2 had greater SO production. Inhibitors of NOS (NMMA) and NOX (DPI) added to the homogenate from mice lacking NOS3 or NOS2 significantly reduced SO production. * = p<0.05 versus WT control, ** = p<0.05 versus genotype matched untreated control.

One general mechanism through which innate immune responses can be stimulated is through reduction-oxidation (redox) signaling. To determine if superoxide (SO, an essential substrate to redox signaling) production was increased in NOS3−/− mice and to determine enzymes regulating its production, the following experiment was performed. Kidneys from MRL/lpr mice with and without functional NOS3 and NOS2 (iNOS) genes were harvested during active disease. Cortical tissue homogenate was analyzed using lucigenin as a marker of SO production. Mice lacking NOS3 more than mice lacking NOS2 had greater cortical SO production (Figure 4). These results suggest that functional eNOS and iNOS both modulate SO production ex vivo. To determine enzyme sources of SO, inhibitors of known SO-producing enzymes were added to the homogenate. SO production was inhibited only by NOS and NADPH oxidase inhibitors (Figure 4) and not inhibitors of xanthine oxidase, mitochondrial electron transport chain complex I, II, or III, cyclooxygenase, cytochrome p450 (Figure S3). These combined data suggest that NOX and uncoupled inducible or neuronal NOS are enzyme sources of SO that are modulated by low levels of eNOS-derived NO.

### Urine MCP1 Levels Increased in Mice Lacking Functional eNOS

**Figure 5:**
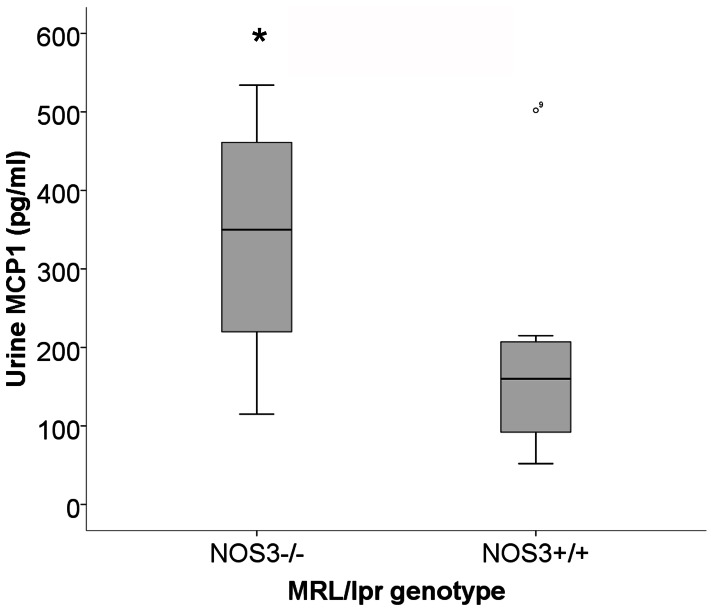
Urine MCP1 is increased in mice lacking NOS3. Urine from NOS3−/− and NOS3+/+ mice with active disease was analyzed for MCP1. Mice lacking NOS3 had significantly greater urine MCP1 levels. * p = 0.019.

Renal cortical SO production, likely inducing redox signaling, was increased in of NOS3−/− mice. One cytokine with redox-mediated transcriptional regulation is MCP1. MCP-1 levels are increased in the urine in lupus nephritis in a prognostic fashion [21]–[23]. It is considered a pathogenic chemokine in lupus nephritis because inhibition of MCP-1 synthesis increases survival and improves nephritis in murine lupus nephritis [24]. MCP-1 is a redox regulated chemokine that is modulated by eNOS-derived NO and stimulated by reactive oxygen species in endothelial cells [25], [26]. We hypothesized that MCP-1 would be redox regulated in this model as well. Therefore, we measured levels of MCP-1 in the urine as a proximal fluid for renal production. To determine potential mechanisms for the protective effect of NOS3 from cellular chemotaxis to the glomerulus, we analyzed the urine of mice from both groups with active disease for MCP1. Mice in the NOS3−/− group had significantly higher MCP1 levels (Figure 5). This suggests that NOS3-derived NO modulated MCP1 production in the setting of lupus nephritis.

## Discussion

This is the first report to date to describe a critical role for eNOS in modulating crescentic, necrotic, hypercellular, tubulointerstitial lesions, and premature death in proliferative lupus nephritis. This work suggests that eNOS prevents induction of innate and cellular immune responses distal to immune complex deposition, possibly via suppressing SO production by NADPH oxidase and uncoupled NOS. While these studies demonstrate that T-cell mitochondrial calcium flux is partially dependent on eNOS, lack of this enzyme is insufficient to affect mitochondrial biogenesis in these cells or the onset and progression of humeral autoimmunity to double-stranded DNA. Critical to this downstream innate immune response is the production of chemokines to attract inflammatory cells into glomerular and tubulointerstitial lesions. This work shows that eNOS is important in the regulation of pathologically important MCP1 in proliferative lupus nephritis.

Endothelial nitric oxide synthase is critical to endothelial physiology. Low levels of NO produced by these cells is classically known for relaxing smooth muscle cells and preventing thrombosis. However, NO produced by endothelial cells is also critical in modulating the effects of inflammatory stimuli such as the production of chemokines and adhesion molecules [27]. There is precedent for the notion that NO regulates MCP1 production in endothelial cells, as inhibition of NO production in cultured human endothelial cells increased MCP1 mRNA and protein expression in association with increased NFκB binding activity, while addition of exogenous NO reduced MCP1 expression and NFκB binding [28].

In many cells, responses to inflammatory stimuli are induced by redox-mediated transcription factors such as AP1 (cJun) and NFκB [29]. This regulation occurs with oxidative modifications of protein thiols critical to enzyme function, much akin to phosphorylation. An example of redox signaling important to inflammation is activation of NFκB through oxidative modification of tyrosine kinases Lck or Src [30]. The MCP1 promoter region contains redox-regulated NFκB, AP-1, and c-Jun response elements [31]. Expression of MCP1 by activated EC can be regulated by reactive oxygen species and can be inhibited by antioxidants [25]. The present study suggests that eNOS regulates SO production and MCP1 production. Whether redox regulation of gene transcription is directly related to MCP1 production in lupus nephritis would be a rational objective of future study.

An earlier study in our laboratory demonstrated that systemic reactive oxygen species (ROS) production was partially dependent on inducible NOS [10]. Because NOS inhibitors prevent SO production from uncoupled NOS, this increased ROS production likely emanated from uncoupled NOS in earlier studies [32]. The current study links NOS to SO production in the renal cortex for the first time. NOS uncoupling occurs when tetrahydrobiopterin (BH_4_), essential to NOS dimerization, is oxidized. This oxidation prevents electron transfer from the reductase to the oxidase domain, resulting in electron donation to oxygen to form SO rather than NO [33]. Studies in mice with transgenic overexpression of eNOS with and without transgenic expression of BH_4_ revealed that the stoichiometry of eNOS and BH_4_ expression or activity regulates the ratio of NO and SO produced. In addition, NOS inhibitors normalized SO production in mice overexpressing eNOS but not BH_4_ [32]. Thus, it is possible that eNOS regulation of SO production also prevents uncoupling of NOS, which is driven by oxidation of BH_4_, potentially in all three isoforms of NOS [34]. The lack of significant effect of NMMA on reducing SO production in the NOS3+/+ mice might indicate less baseline uncoupling of NOS in these mice and highlights the importance of NOS3 in preventing SO production via both NOX and NOS. Another possibility is that superoxide dismutase (SOD) is expressed in higher levels in NOS3+/+ endothelial cells. SOD itself can be regulated by NO in endothelial cells [35]. Greater expression of SOD in NOS3+/+ mice would lead to accelerated metabolism of SO to H_2_O_2_, which is not measured by the lucigenin assay. The source of ROS that might lead to BH_4_ oxidation in lupus nephritis is not clear; however, the present study strongly suggests that NADPH oxidase is an important catalyst of SO production in proliferative lupus nephritis.

Several NADPH oxidases (NOXs) are expressed in endothelial cells. NOX1 and NOX4 are most highly expressed in renal tissue [36], specifically in vessels, glomeruli, podocytes, and tubular cells. How SO production is induced in lupus nephritis is not well described. Given that the protective effect of eNOS appears to be important after immune complex deposition, one might postulate that immune complexes play a role in this process. Supporting this notion, endothelial cell Fc receptors that are activated by immune complexes signal for ROS production in a NOX-dependent fashion [37], [38]. Whether this is the operative mechanism in lupus nephritis would be the object of future research.

This study demonstrates that both iNOS- and eNOS-derived NO modulate SO production, as lack of either NOS isoform leads to increased SO production. There is precedent in the literature for NO-mediated regulation of NADPH oxidase NOX2 and NOX1. For instance, the p47phox organizer subunit of NOX2 is nitrosylated by NO, resulting in reduced activity and generation of SO [39]. In addition, NOX1 transcription is regulated by NO in mesangial cells [40]. Thus, the duality of NOS is revealed in its regulation of NOX SO production in its coupled form and production of SO in its uncoupled form.

The importance of eNOS in vascular physiology was demonstrated in non-autoimmune murine models. For instance, mice lacking eNOS have reduced vasculogenesis in the presence of limb ischemia [41]. Relevant to renal physiology, mice lacking eNOS have significantly increased blood pressure and plasma renin concentrations [42]. While this phenomenon might accelerate renal failure, the pathology observed in the mice in this study was not consistent with hypertensive renal crisis. These studies are relevant to the pathophysiology of human disease in that eNOS expression is reduced in human proliferative lupus nephritis. Whether eNOS expression increases after successful treatment is not known. In addition, endothelial dysfunction is much more prevalent in SLE without nephritis [43]. Lower eNOS expression in this setting could predispose redox-mediated innate immune responses in other tissues with immune complex deposition, but this hypothesis was not tested in this study.

Our findings are consistent with earlier reports of increased vasculitis in MRL/lpr mice lacking eNOS [28]. However, in the study by Schoeb et al., no differences in glomerulonephritis or mortality were observed in the NOS3+/+ and NOS3−/− mice. While it is open to interpretation, these differences may be explained by differences in methodology between the two studies. First, mice in this study were studied at a much younger age due to the accelerated mortality in the NOS3−/− mice in our study. When 12 and 16 week old mice in the Schoeb study were examined, there was a trend to greater glomerulonephritis in the NOS3−/− mice. The differences in mortality between this and the Schoeb study may be indicative of another important difference in the studies. In the current study, the NOS3 heterozygotes were generated using speed congenics after nine back crosses to the MRL/lpr mice with periodic back crosses to MRL/lpr mice to prevent genetic drift. The confirmed presence of 15 different known MRL/lpr susceptibility loci in the present study ensured a strong SLE background and likely accelerated the rate of onset of glomerulonephritis relative to the rate observed in the Schoeb study. One could argue that complete ablation of eNOS is an artificial construct that does not reflect the pathophysiology of human disease. However, eNOS expression is reduced in human proliferative nephritis as well [6]. In addition, this model system highlights the critical role for eNOS in regulating SO production. Thus, even if eNOS expression is not completely eliminated in human lupus nephritis as in this model, increasing the activity of eNOS could still be a valid therapeutic target. The focus on early nephritis in the present study compared to the Schoeb study could be construed as a limitation. However, this focus enhances the clinical relevance of the findings, as early disease represents that during which clinicians typically begin induction therapy to prevent irreversible glomerular and tubulointerstitial fibrosis. This would, therefore, be an ideal phase of disease or increasing eNOS function as a therapeutic target.

**Figure 6:**
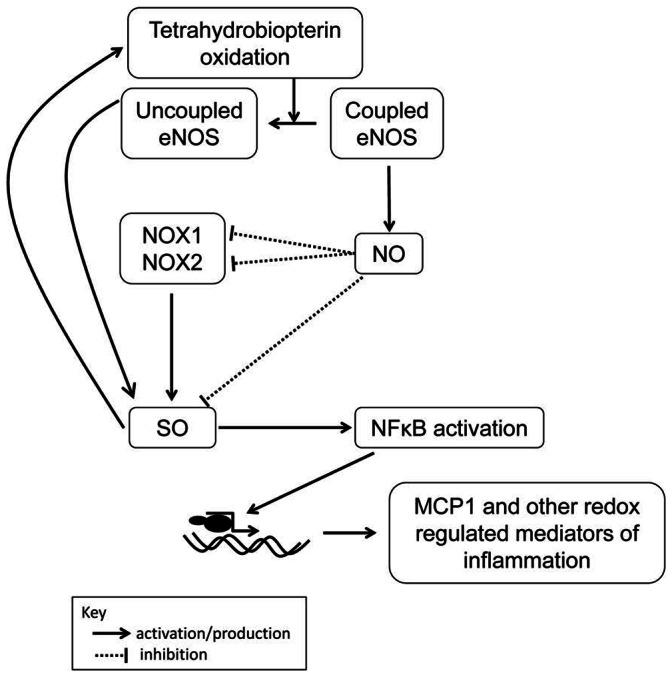
Proposed mechanism for dual role of eNOS as regulator of and producer of superoxide (SO) production. Coupled eNOS produces NO that can modulate SO production by NOX1, 2, and 4 via changes in transcription (NOX1) and enzyme activity (NOX2) and scavenge SO directly. However, these NOX enzymes can produce SO themselves, which in turn can oxidize BH4, leading to uncoupling of NOS. This, in turn, leads to SO production. SO, metabolized to hydrogen peroxide, can activate NFκB that can itself increase the transcription of redox-regulated inflammatory mediators such as MCP1.

This study offers several important and novel findings. It demonstrates a critical role for NOS activity in regulating SO production and MCP1 production in proliferative lupus nephritis. It highlights the complex and antithetic roles of coupled and uncoupled NOS in both regulating and producing SO. A postulated pathway describing these roles was derived from the current study and pathways described in the literature (Figure 6). This study also demonstrates that eNOS is critical in modulating the development of clinically relevant necrotic and crescentic glomerular lesions as well as premature death. The results of this study give a rational basis for the study of therapeutics designed to increase eNOS expression, activity, or function to improve outcomes in proliferative lupus nephritis.

## Supporting Information

Figure S1 **Glomerular immunostain intensities of IgG and C3 in NOS3−/− and NOS3+/+ mice are no different.** Snap frozen cortical tissue from NOS3−/− (n = 10) and NOS3+/+ (n = 8) mice was cryosectioned and immunostained for C3 (black bars) and IgG (white bars). Results were reported in mean intensity for each of ten glomeruli examined for each mouse. IgG and C3 staining intensity was no different between genotypes (p>0.05 for all comparisons).
(TIF)

Figure S2 **Serum anti-doubles stranded DNA antibody (dsDNA) levels are no different between MRL/lpr and MRL/lpr NOS3−/− mice.** Serum from MRL/lpr mice (n = 15) and MRL/lpr NOS3−/− mice (n = 23) was analyzed for dsDNA in dilutions between 100 and 800. Results are reported as optical density (OD). No differences (p>0.05) were observed between MRL/lpr mice (NOS3+/+, solid line) and NOSe−/− mice (dotted line) at any of the dilutions.
(TIF)

Figure S3 **Renal cortical SO production in NOS3−/− and NOS2−/− mice is not affected by inhibitors of cyclooxygenase 1 and 2 (Indo and MFA), cytochrome p450 (NADPH), xanthine oxidase (Allo), and mitochondrial electron transport chain complexes I (ROT), II (TTFA), and III (Ant A).** Renal cortical tissue from NOS3−/−, NOS2−/− and wild-type littermates (WT) was examined for SO production in the presence of inhibitors of known enzyme sources of SO production. Inhibitors had no effect on SO production (p>0.05 for all comparisons within genotype but between inhibitor and no treatment (none)).
(TIF)
