# Supplementary figures and images for: Endothelial Nitric Oxide Synthase Reduces Crescentic and Necrotic Glomerular Lesions, Reactive Oxygen Production, and MCP1 Production in Murine Lupus Nephritis
Source: PLoS One. 2013 May 31;8(5):e64650. doi: 10.1371/journal.pone.0064650 (PMC3669382; doi:10.1371/journal.pone.0064650)

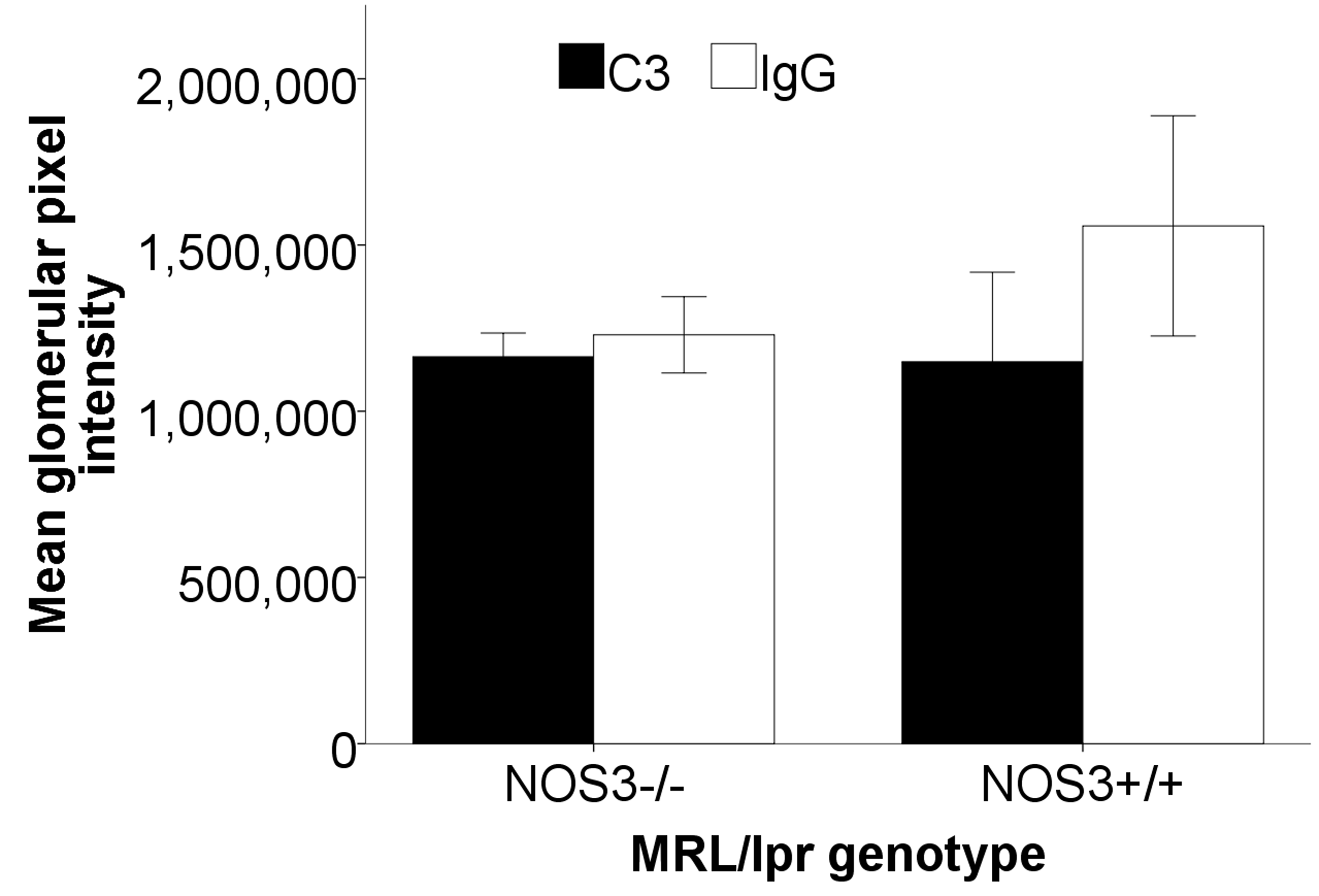

Supplement: Figure S1 — Glomerular immunostain intensities of IgG and C3 in NOS3−/− and NOS3+/+ mice are no different. Snap frozen cortical tissue from NOS3−/− (n = 10) and NOS3+/+ (n = 8) mice was cryosectioned and immunostained for C3 (black bars) and IgG (white bars). Results were reported in mean intensity for each of ten glomeruli examined for each mouse. IgG and C3 staining intensity was no different between genotypes (p>0.05 for all comparisons). (TIF) [file pone.0064650.s001.tif]

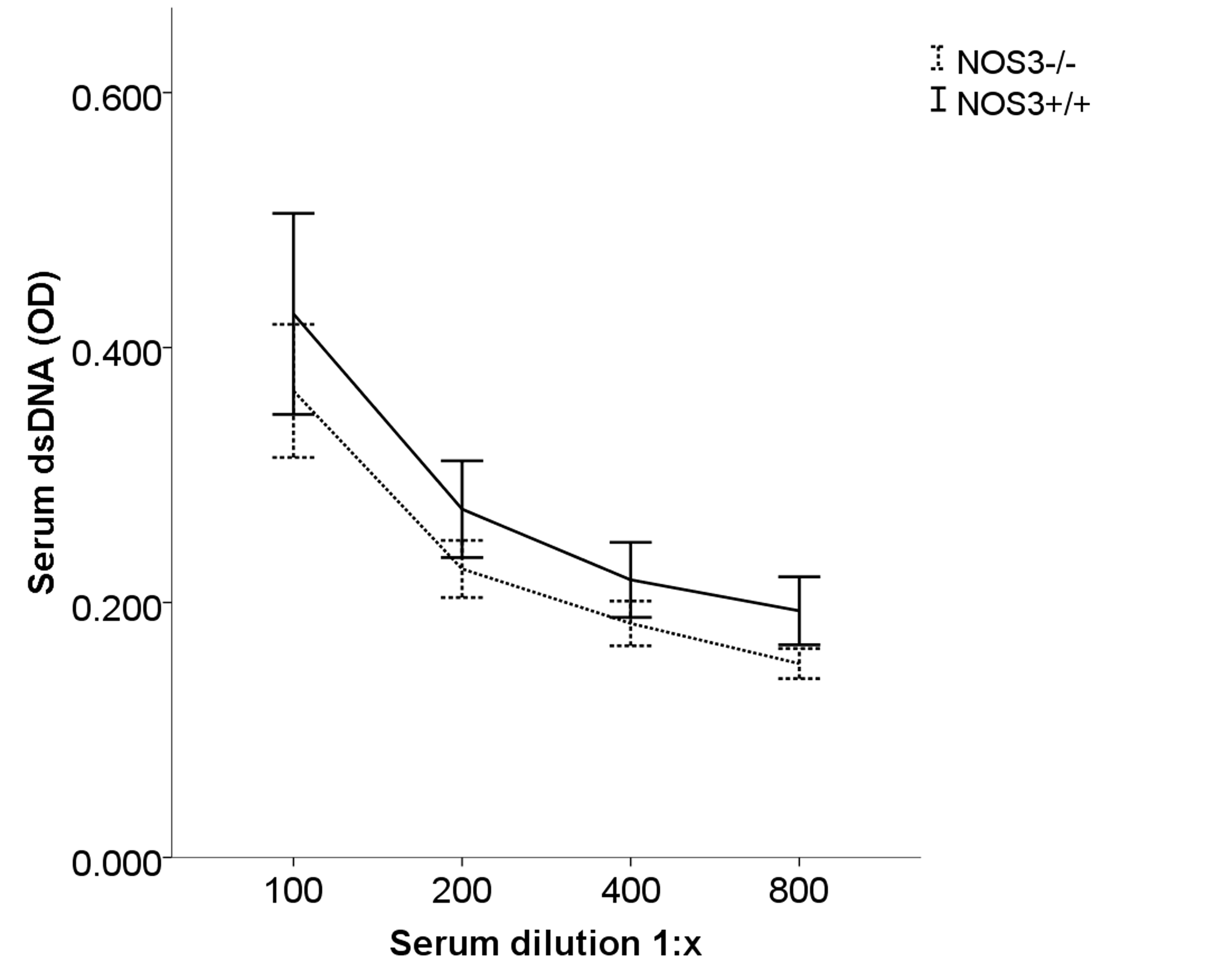

Supplement: Figure S2 — Serum anti-doubles stranded DNA antibody (dsDNA) levels are no different between MRL/lpr and MRL/lpr NOS3−/− mice. Serum from MRL/lpr mice (n = 15) and MRL/lpr NOS3−/− mice (n = 23) was analyzed for dsDNA in dilutions between 100 and 800. Results are reported as optical density (OD). No differences (p>0.05) were observed between MRL/lpr mice (NOS3+/+, solid line) and NOSe−/− mice (dotted line) at any of the dilutions. (TIF) [file pone.0064650.s002.tif]

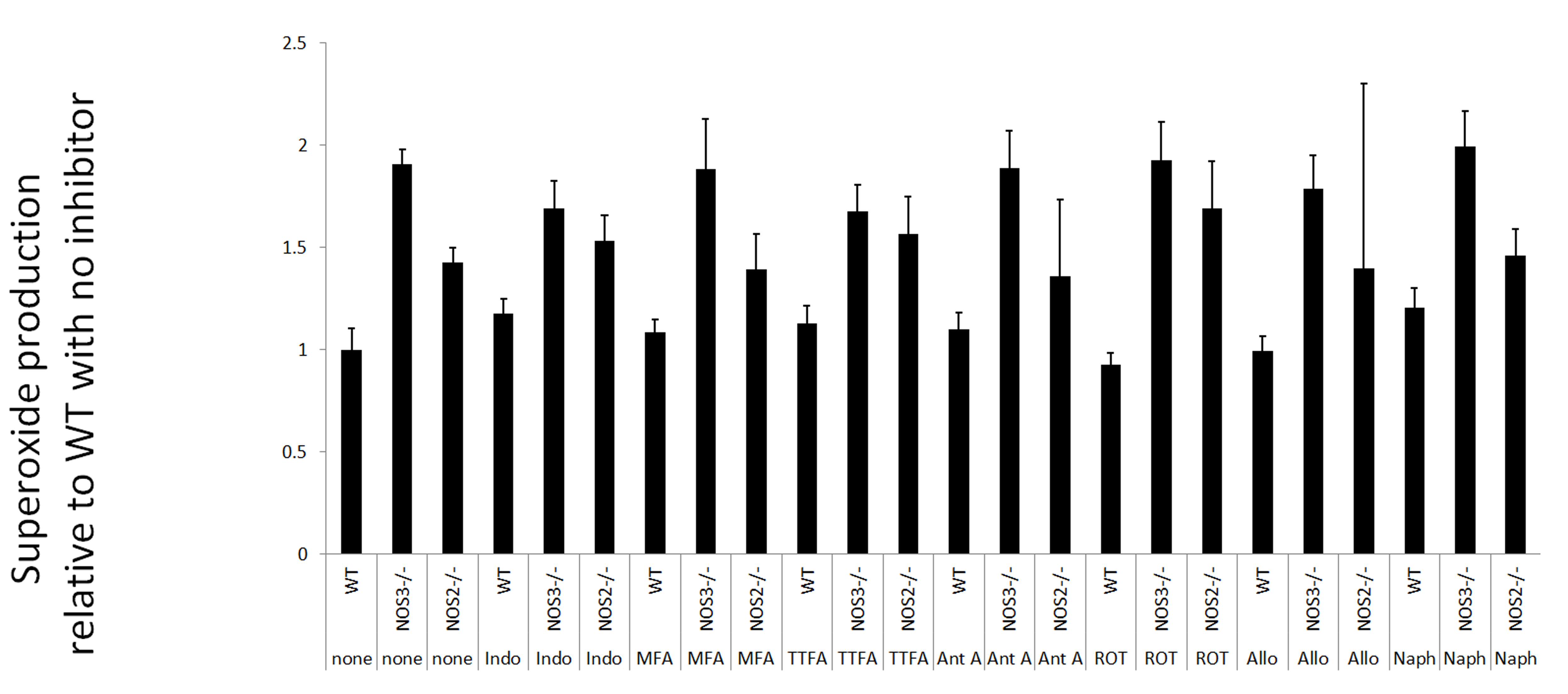

Supplement: Figure S3 — Renal cortical SO production in NOS3−/− and NOS2−/− mice is not affected by inhibitors of cyclooxygenase 1 and 2 (Indo and MFA), cytochrome p450 (NADPH), xanthine oxidase (Allo), and mitochondrial electron transport chain complexes I (ROT), II (TTFA), and III (Ant A). Renal cortical tissue from NOS3−/−, NOS2−/− and wild-type littermates (WT) was examined for SO production in the presence of inhibitors of known enzyme sources of SO production. Inhibitors had no effect on SO production (p>0.05 for all comparisons within genotype but between inhibitor and no treatment (none)). (TIF) [file pone.0064650.s003.tif]
